# Appraisal of primary health care services in Federal Capital Territory, Abuja, Nigeria: how committed are the health workers?

**DOI:** 10.11604/pamj.2017.28.134.12444

**Published:** 2017-10-11

**Authors:** Taiwo Akinyode Obembe, Kayode Omoniyi Osungbade, Christianah Ibrahim

**Affiliations:** 1 Department of Health Policy and Management, Faculty of Public Health, University of Ibadan, Ibadan, Nigeria; 2 School of Public Health, Faculty of Health Sciences, University of Witwatersrand, Johannesburg, South Africa; 3 Department of Health and Human Services, Federal Capital Territory Administration, Abuja, Nigeria

**Keywords:** Primary health care, millennium development goals, basic health service scheme, selective primary health care, health-for-all

## Abstract

**Introduction:**

The primary health care model was declared as the appropriate strategy for ensuring health-for-all. However up till date, very few studies have assessed the services provided by primary health centres in terms of its basic components. This study aimed to appraise health services provided and to estimate the commitment of the health workers in selected primary health care centres within Abuja Nigeria.

**Methods:**

A cross sectional study was utilized to obtain information from 642 health workers across 6 area councils of the Federal Capital Territory, Nigeria. Data collection was performed using pre-tested, structured, interviewer-administered questionnaires and data were analyzed at 95% level of significance using SPSS version 17.0.

**Results:**

Our study participants were largely females (58.6%), Christians (63.2%) and aged 30-39 years (40.0%). Health services offered in centres were adequate in all components of PHC except for mental health (23.7%) and care of the elderly (43.0%). Conduct of home visits was least practiced by health workers (83.8%) compared to the use of patient appointments (96.4%) and conducting staff outreach activities (94.9%). Commitment was three times more likely when service was related to health promotion and education (OR = 2.52; CI = 1.23-5.18); nutrition education (OR = 3.13; CI = 1.13-8.68).

**Conclusion:**

Health workers in primary health centres of the federal capital territory still provide sub-optimal services with respect to mental health and care of elderly. Concerted efforts and unrelenting political will to strengthen mental and geriatric health components are recommended.

## Introduction

From pre-colonial times, the health system has garnered much attention in terms of structure and services. The journey to establishing an equitable health system in Nigeria began in 1960 with the 10-year development plan (1946-1956) [1], also referred to as the "Decade of Development" [2]. This was followed in turn by the Second Colonial Development Plan (1956-1962); First National Development Plan (1962-1968); Second National Development Plan (1970-1975); Third National Development Plan (1975-1980); Fourth National Development Plan (1981-1985) and the most recent Five year Strategic Plan (2004-2008) [2]. However, before the Fourth National Development Plan was introduced in Nigeria, primary health care (PHC) was embraced as a key strategy for attaining health-for-all by the year 2000 [3]. This was in 1978 agreed by 134 countries as 'a foremost human right identified as pivotal to delivering health to all by 2000 (pp. 1) [4]. According to Barbara Starfield, it was described as, "the provision of first contact, person-focused, ongoing care over time that meets the health-related needs of people, referring (to hospital) only those problems too uncommon to maintain competence and coordinate care when people receive services at other levels of care" [5]. Primary health care model and strategy codified as Comprehensive Primary Health Care (CPHC) was launched with the aim of achieving Health-for-All by the year 2000 [6]; but then reviewed and with the institution of Development Goals (to assist in facilitating its success), extended to 2015 and most recently the post-2015 development goals [3]. The Comprehensive Primary Health Care (CPHC) today has enjoyed several accolades and likewise, a number of criticisms. Popular criticisms of CPHC in the literature range from scholars stating the strategy represents "poor treatment for poor people" since it was based on low-cost and low technology choices [7] to skeptics who believe that it was more relevant to developing countries [6, 8, 9]. Others have raised concerns with its cost-effectiveness and the feasibility of its implementation [10]. Nigeria, as a signatory and member country in the declaration of Alma-Ata took its first proactive step towards ensuring Health-For-All initiative with the review of its 3^rd^ National Development Plan.

**Figure 1:**
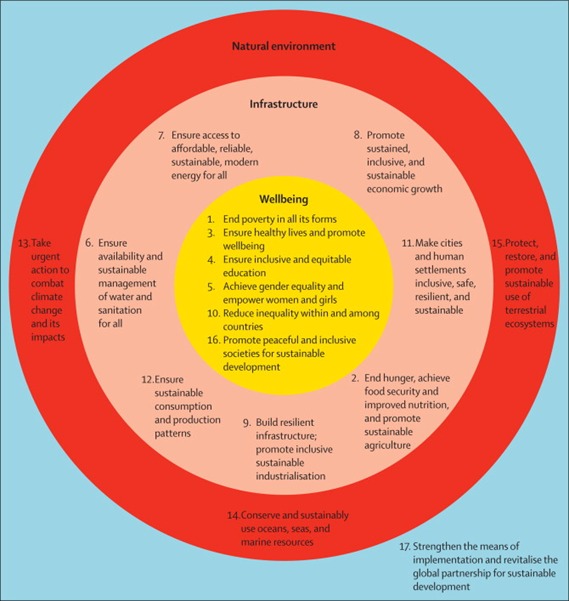
Framework for examining between sustainable development goals *(Reproduced with permission)*

This led to the development and introduction of the 4^th^ National Development Plan, which had as its primary focus the need to adopt more seriously the subject of preventive health services. This policy document upheld the enactment of Basic Health Service Scheme (BHSS) [11], which was to provide health care services in 3 tiers of facilities specifically: "a) Comprehensive Health Centres (CHC) to serve communities of more than 20,000 people; b) Primary Health Centers (PHC) to serve communities of 5000 to 20,000 persons; and c) Health Clinics (HC) to serve 2000 to 5000 persons" (pp. 55) [2]. Even though, the realization to this laudable initiative has been impeded by several systemic factors, Dungy (1979) highlights fundamental principles worthy of emulation by developed countries in the Basic Health Service Scheme which include: a) Use of non-physician providers; b) Use of rural and distant sites for training health professionals; c) Maintenance of medical records by the consumers/patients (i.e. use of home based records instead of hospital based records) [12]. Nevertheless, the gaps in the concept of CPHC led to its review and subsequent adoption of "Selective Primary Health Care" (SPHC) by some member countries. An argument against the former model was that it's vision to ensure total population coverage was too idealistic, high-priced and impracticable [6]. Unlike the CPHC, the selective PHC focuses public health issues such as growth monitoring, oral rehydration, breastfeeding and immunization [6]. Today, the policy and model of PHC has been reviewed yet again in what is addressed as the post 2015 agenda, now supported by "Sustainable Development Goals" (SDGs) instead of the former "Millennium Development Goals" (MDGs) [13, 14]. However, unlike the MDGs that have three out of eight health related goals, there is only 1 health goal in the proposed SDGs (Figure 1) [15], which already raises issues of concern with regards to upgrading of health systems and their abilities to provide equitable care, particularly to the grassroots. In the light of this, it is prudent to evaluate the services of CPHC that have been operational since 1978. Abdulraheem and colleagues (2012) recommend an operational strategy involving a comprehensive baseline survey to collect information about health status, socio-demographic variables, civic amenities, existing health facilities as well as the attitudes and beliefs of the target population towards PHC services [8]. This study thus sought to appraise health care services and assess the level of commitment to these services within selected primary health centres in the Federal Capital territory, Nigeria.

## Methods

The Federal Capital Territory (FCT) in Abuja, Nigeria was the study site for this research. Abuja came into existence by virtue of the Federal Capital Territory Act, of 1976. It spreads over a land mass area of approximately 7,315 square kilometers (sq.km), of which the definite city occupies 275.3 square kilometers (sq.km). It is made up of 6 Area Councils-Municipal, Abaji, Bwari, Gwagwalada, Kuje and Kwali. The inhabitants are mostly Christians and Muslims, though traditional worshippers also abound with over 60% of its populace still living in the rural areas across the 6 area councils [16]. Earliest FCT indigenes were of the Kwa language group that was predominantly found around the Niger-Benue confluence while other groups in the FCT include Bassa, Gade, Gwandara, Koro and Ganagana, all of which have deep affiliation with the Kwa language groups dominant in the present middle belt area. The main occupation of indigenes is subsistence farming, but many are engaged in wood and craftwork (common amongst the Gbagyis), iron work (common amongst the Ganaganas), and cloth weaving (common amongst the women) [16]. Minimum sample size of 426 respondents was calculated using 5% level of significance and 50.0% as the proportion of health workers committed to providing PHC services at their respective centres at 95% level of significance. This sample size was estimated to account also for 10% non-response rate and for inadequately filled questionnaires. We used 50.0% as our desired proportion for response distribution in order to obtain the largest sample size [17]. A total sampling technique was used to recruit all eligible 642 participants (that consented to participate) from 694 eligible health workers across the 6 area councils of the Federal Capital Territory (Response rate of 92.5%) in a cross-sectional study design.

Eligibility to participate in the study was determined by occupational class and duration in area council. Participants had to be at least junior community extension workers (JCHEW) with a minimum work experience of at least 3 months in the area council while we excluded volunteers and social workers at the time of the study. Data were collected using interviewer-administered semi-structured questionnaire that was pre-tested in a primary health care facility (in Suleja local government of Niger state) similar to the proposed study site. Our independent variables for the bivariate analysis was availability of a PHC service while commitment of staff was taken as the dependent variable. Availability of a service was self-reported and verified by evidence of case records of patients attended to within the last month before the data collection exercise. For every service that was checked, entries were inputted as a binary variable (1 = Yes, 0 = No). Commitment was assessed using 14 item questionnaire to measure service commitment with regards to all the components of primary health care [18]. Scores 0-6.9 and scores above 7 were graded as "not committed" and "committed" respectively. A Cronbach's Alpha of 0.83 was obtained from the reliability assessment of the instrument before its final administration in our study site. Data on sociodemographic characteristics of all the workers at the primary health centres were obtained and recorded. Statistical analysis was performed using Statistical Package for the Social Sciences (SPSS) version 17.0 (Chicago Il, USA) after the data were coded, checked. Descriptive statistics such as frequencies, and proportions were used to summarize variables. Chi-square tests were used to identify associations between categorical variables at 5% level of significance. Ethical approval was obtained from Federal Capital Territory Health Research Committee. Both verbal and written informed consents were obtained from the respondents. The privacy and confidentiality of respondents´ data were assured.

## Results

**Figure 2:**
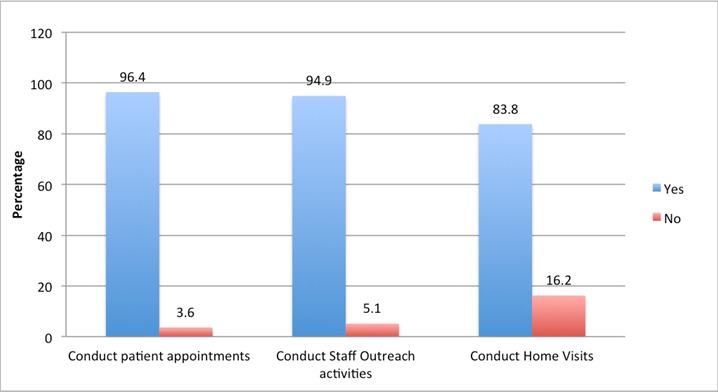
Proportion of workers that conduct appointments, outreaches and home visits

**Figure 3:**
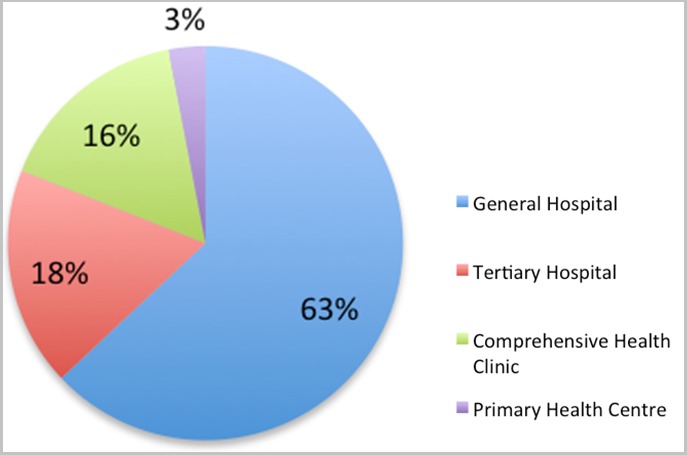
Referral centres used by primary health care centres

The study participants were largely females (58.6%), Christians (63.2%) and aged 30-39 years (40.0%). Most were non-indigenes (59.2%) and married (73.4%). The predominant local language spoken in the study area was Hausa (35.4%) and the leading ethnic group were the Hausas (41.6%). Majority of the staff had completed some post-secondary education (89.3%) (Table 1). Majority of the respondents were recruited were from the primary health centres (53.7%) and health clinics (23.4%). The most common service that participants attested to providing was, "treatment of common diseases/minor injuries" (98.0%). Other services were listed and satisfactorily offered in the centres except for the "care of the elderly" (43.0%) and mental health (23.7%) (Table 2). Use of appointment scheduling to manage patients' ailments was practiced by most (96.4%) of the respondents (Figure 2). Commonest reason provided for scheduling the appointments was for follow-up on continuity and effectiveness (66.8%) of treatment (Table 2). Conduct of home visits to supplement management of ailments was least practiced by health workers (83.8%) compared to the use of patient appointments (96.4%) and conducting staff outreach activities (94.9%) (Figure 2). In most cases, patients needing specialist care were referred to General Hospitals (63.0%) followed by tertiary health facilities (18.0%) (Figure 3).

**Table 1:** Socio-demographic characteristics of respondents (N = 642)

		Frequency (n)	Percentage (%)
**Age group (years)**	<30	221	34.4
	30-39	257	40.0
	40-49	102	15.9
	≥50	62	9.7
**Sex**	Male	266	41.4
	Female	376	58.6
**Ethnicity**	Hausa	267	41.6
	Igbo	170	26.5
	Yoruba	205	31.9
**Religion**	Christianity	406	63.2
	Islam	234	36.4
	Traditional	2	0.4
**Marital Status**	Never Married	171	26.6
	Ever Married	471	73.4
**Community Status**	Indigene	262	40.8
	Non-Indigene	380	59.2
**Local Languages Spoken**	Hausa only speaking	227	35.4
	Nga only speaking	99	15.4
	>1 local Language i.e. Hausa and Nga	116	18.1
	Others+	200	31.1
**Highest Education**	Primary	10	1.5
	Secondary	573	89.3
	Tertiary	59	9.2
**Occupational Characteristics** (Years of Experience)			
	<1year	17	2.6
	1 - <5years	254	39.6
	5 - <10 years	192	29.9
	10 - < 15 years	61	9.5
	15 - <20	57	8.9
	≥20	61	9.5
**Working years in present health facility**	<1year	258	40.2
	1 - <5years	234	36.4
	5 - <10 years	64	10.0
	10 - < 15 years	29	4.5
	15 - <20	28	4.4
	≥20	29	4.5

+ Other local languages include other Chadic languages such as: goemais, Mwaghavuls, Boles, Ngizims, Bades and Bachamas

**Table 2:** Types of primary healthcare facilities and services provided

Type of Health Facility (n=642)	Frequency (n)	Percentage (%)
Comprehensive Health Centre (CHC)	115	17.9
Primary health centre	345	53.7
Health clinic	150	23.4
Health post	32	5.0
**Services offered by health facility (n=642)**		
Treatment of common diseases/minor injuries	629	98.0
Immunization against common childhood diseases	626	97.5
Ante natal care	621	96.7
Health promotion and education	607	94.5
Oral Rehydration Therapy	590	91.9
Provision of essential drugs	586	91.3
Nutrition education	585	91.1
Family planning services	578	90.0
In-patient deliveries	446	69.5
Post-natal care	528	82.2
Adult care	434	67.6
Oral health care	413	64.3
Emergency care	404	62.9
Care of the elderly	276	43.0
Mental health care	152	23.7
**Reasons for Appointment** **(n=619)+**		
Follow up for effectiveness & continuity of treatment	414	66.8
Ante natal care revisit	50	8.1
Post-natal care follow up	47	7.6
Follow up of immunization services	35	5.7
Follow up for family planning	25	4.0
Elective procedure	22	3.6
Proper care & diagnosis	19	3.1
Laboratory results	7	1.1

+ Participants that answered ‘yes’ to carrying out the activity 619 (96.4%) in Figure 2

Outreaches centered on activities such as exclusive breastfeeding, awareness of existing PHC health facilities within the community and MDGs. Of the 609 respondents that have outreaches, immunization was the most conducted activity (99.0%) followed by activities that exhibit importance of health promotion and education (82.9%) (Table 3). Five hundred and thirty-eight (83.8%) respondents claimed to go on home visits (Figure 2). Among these respondents the most common reasons for home visits were to check on mothers who failed to show up with their child (ren) for immunization (87.9%) and also to follow up on patients that had been recently discharged from the facility (83.3%) (Table 3). Overall, 81.9% of the respondents were committed to providing services related to primary health care to the community. Commitment to services at the PHCs were only significant with Health Promotion and Education services (p = 0.010), Nutrition Education (p = 0.025), Oral Rehydration Therapy (p < 0.000), Immunization services (p = 0.015) and Treatment of common disease/minor injury (p = 0.002) (Table 4). Commitment was three times more likely when service was related to health promotion and education (OR = 2.52; CI = 1.23-5.18); nutrition education (OR = 3.13; CI = 1.13-8.68). It was also observed that commitment was almost 4 times likely when it was related to immunization (OR = 3.53; CI = 1.25-10.00) and 5 times more likely when service was related to treatment of common disease/minor injury (OR = 5.68; CI = 1.80-17.95). (Table 4).

**Table 3:** Types of outreaches conducted and respondents’ reasons for conducting home visits

Self-reported variables	Frequency (n)	Percentage (%)
**Activities carried out in outreaches** **(n=609)+**		
Immunization	596	99.0
Health promotion and education	505	82.9
Awareness on environmental sanitation	496	81.4
Follow up of TBAs	336	55.2
HIV/AIDS awareness	11	1.8
House-to-House inspection	4	0.7
Sex education	2	0.3
Community dialogue	2	0.3
Disease surveillance	2	0.3
Awareness of existing PHC health facility within the community	1	0.2
Exclusive Breast Feeding	1	0.2
MDG outreach	1	0.2
**Purpose of home visit (N = 538)+**		
Failure of mother to bring child for immunization	473	87.9
Follow up after discharge from facility	448	83.3
Defaulters on TB DOTS treatment	3	0.6
Child’s growth monitoring	1	0.2
Supervision of nutritional food demonstration & nutritional counseling	4	0.7
Failure of patient to turn up for follow up	7	1.3
Ambulatory patients	1	0.2
Post-natal care	3	0.6
Ante-natal follow up	7	1.3

+ Participants that answered ‘yes’ to carrying out outreaches 609 (94.9%); home visits 538 (83.8%) in Figure 2

**Table 4:** Bivariate associations between services rendered in PHCs and commitment of health workers at health centres (N = 642)

	Committed	Not Committed	X^2^	P-value	Odds Ratio (95% CI)
**Health Promotion and Education**					
Yes	503 (82.9)	104(17.1)	6.58	0.010+	2.52 (1.23-5.18)
**Nutrition education**					
Yes	517(82.5)	110(17.5)	4.99	0.025+	3.13 (1.13-8.68)
**Antenatal care**					
Yes	516(82.4)	110(17.6)	4.19	0.041	2.81(1.03-7.66)
**In-patient deliveries**					
Yes	368(81.6)	83 (18.4)	0.115	0.735	0.93(0.59-1.44)
**Post-natal care**					
Yes	438 (83.0)	90 (17.0)	2.10	0.147	1.44 (0.88-2.35)
**Oral Rehydration therapy**					
Yes	496(84.2)	93 (15.8)	25.03	0.000+	4.09 (2.29-7.32)
**Family planning services**					
Yes	476(81.2)	110(18.8)	2.24	0.134	0.52 (0.22-1.23)
**Immunization against common childhood diseases**					
Yes	518 (82.5)	110 (17.5)	5.94	0.015+	3.53(1.25-10.00)
**Treatment of common disease/minor injury**					
Yes	521(82.6)	110 (17.4)	10.06	0.002+	5.68(1.80-17.95)
**Provision of essential drugs**					
Yes	482 (81.7)	108 (18.3)	0.28	0.600	0.81 (0.37-1.76)
**Oral health care**					
Yes	340(83.5)	67(16.5)	1.94	0.164	1.34 (0.89-2.01)
**Mental Health care**					
Yes	119(78.3)	33(21.7)	1.78	0.182	0.74 (0.47-1.15)
**Adult care**					
Yes	359(81.9)	79(18.1)	0.001	0.975	1.01 (0.65-1.55)
**Care of the Elderly**					
Yes	226(80.1)	56(19.9)	1.09	0.297	0.81 (0.54-1.21)
**Emergency Care**					
Yes	343(82.1)	75(17.9)	0.01	0.910	1.02 (0.67-1.56)

+ - Significant Associations

## Discussion

Majority of our participants were recruited from the primary health centres. This is not surprising since a minimum number of four primary health centres are expected to feed into each comprehensive health centre as stipulated by the National Primary Health Care Development Agency (NPHCDA) [19]. Despite the relative availability of health personnel at the primary care centres, dispensation of primary care services at wards has remained sub-optimal and below acceptable standards with uneven distribution of health personnel [20] across the different health facilities, as corroborated also by our study findings. Neglected and often overlooked supply side factors such as training opportunities, career development prospects, health workers'preferences may be important factors implicated in the dismal availability of health workers in the health posts [21]. Moreover, the mere fact that health centers typically have on ground a full-time graduate doctor unlike in health posts [22], may serve as a deterrent for retaining sufficient health workers and ultimately patronage by health care consumers. The services rendered in the health facilities could be perceived appropriate in terms of Primary Health Care for all basic components except for Mental Care and Care of the Elderly. Despite global calls by World Health Organization on the need to prioritize mental care services at the primary care level [23], attention to diagnosis and treatment of mental disorders has suffered great limitations in Nigeria. The documentation of associations between severe mental illnesses and physical disorders or increased odds of reporting difficulties in accessing care [24] makes neglect of mental disorders particularly worrisome. Poor attention to mental disorders could be as a result of a severe shortage of psychiatrists (with an estimated 150 psychiatrists in a country of 160 million) [25] or deeply rooted traditional and socio-cultural beliefs that mental disorders are largely as a result of supernatural forces, with much emphasis on supernatural disruptions [26]. Thornicroft and colleagues (2010) advocate the need for a community-oriented mental health care based on the balanced care model to bridge gaps in mental care access and treatment stressing that this form of care not only sustains connection with close family ties but also preserves fundamental human rights to provide needed equitable care in the "least restrictive environment" [27]. Even though caregivers are favorably disposed towards caring for the elderly relatively [28, 29], institutional care is still largely a rejected phenomenon [29] in most African settings, which is corroborated by our study findings. The acknowledgement of appointment scheduling to follow up cases is encouraging, as appointment scheduling has been identified to lie at the intersection of timely access to health care services and efficiency [30]. Agada and colleagues (2015) substantiate the realization and adoption on the need for effective appointment scheduling by advocating for a mixed block appointment rather than existing single block appointment systems [31]. Nevertheless, in an out-patient department in Lagos, Nigeria, the SMS-based (text-message) appointment scheduling system is a welcome idea that has linked with better health outcomes [32]. Even in the face of a favorably disposed clientele, considerable research is still required to rigorously determine the pros and cons of various methods of scheduling appointments [33] and to determine which is suitable and compatible to our local context, especially in remote areas where PHCs are more likely to be situated.

The most common reason for home visits, "*defaulter tracing*" which aims to ensure completion of immunization supports findings by Onyiriuka (2005) in Edo State [34]. The importance and relevance of home visits is critical to the success of immunization programs [35]. The poor follow up/home visits for defaulters on Tuberculosis directly observed treatment, short course (TB DOTS) treatment programs at these health centres, is however worrisome because, the onus for patients' adherence to treatment lies with the health care workers [36]. This poor follow up depicts a degree of nonchalance that can only fuel an increased prevalence of TB drug-resistant cases and ultimately a failure of intervention programs. Lingering myths that associate Tuberculosis with impoverishment, sexual promiscuity and HIV/AIDs [37] could be reasons responsible for poor follow up by health workers to defaulters on TB DOTS program. Negative attitudes as a result of improper training of health care workers on tuberculosis is yet another major flaw in minimizing TB treatment defaults [36]. The preponderance of female health workers in our study could reasonably explain the basis for poor follow-up as level of awareness among males is documented to be significantly more than females [38]. The possibilities of poor follow up for defaulters cannot be overlooked since avoidance of stigma may encourage the concealment of TB cases amongst family members [38]. Services significantly linked with commitment by health workers across the health centres include health promotion & education, nutrition education, immunization, treatment of common diseases/minor injury and oral rehydration therapy (ORT). Satisfactory availability of nutrition education services is substantiated by Adeyemi & Oyewole (2012) but is in contrast with Adie (2014) in which a nutrition officer in an in-depth interview expressed severe dissatisfaction with facilities and an urgent need of a nutrition demonstration room [39, 40]. Findings from Omuemu and colleagues also validate availability of oral rehydration therapy. Notwithstanding the findings of this research, there were a number of limitations, just like in cross-sectional studies; causal relationships could not be inferred between variables. Also, our study conducted an overall appraisal of services and commitment rendered across the sample population rather than disaggregating availability of services in terms of type of facility or local government area or cadre of health worker. Moreover, our study was not able to determine whether poor demand-side factors accounted for sub-optimal availability of some basic services (such as the Mental Care and Care of the Elderly) or if in fact supply-side factors were largely responsible. Our study could have inquired exactly how the appointments were scheduled and duration or interval of scheduling. The fact that this was not sought makes it impossible to compare what is being practiced in our local setting with international standards or practices. Furthermore, it is desirable if studies could be conducted to assess and objectively estimate the effects of the appointment scheduling on patient compliance, outcomes and access to care in the surveyed centres.

## Conclusion

Findings from our study show that most services are offered at primary health centres except for mental health care and care of the elderly. Use of appointment scheduling, home visits and staff outreach services were also utilised to enhance management of cases satisfactorily. However commitment of workers was significantly associated only with health promotion & education services, nutrition education, oral rehydration therapy, immunization against common childhood services and treatment of common diseases. Concerted efforts through PHC focused interventions with sustained political will are advocated to improve the availability of other components of PHC services. The PHC model should not be considered obsolete even though the Millennium Development Goals were not achieved by 2015 as it offers promising opportunities for achieving the Health-for-All initiative.

### What is known about this topic

- There is poor utilisation of primary health care facilities in Nigeria;
- Education is positively associated with utilisation of primary health care services;
- Maternal and child health, prompt attention, and appropriate outpatient are some reasons provided by patients that attract respondents to use PHC services.

### What this study adds

- Services offered in primary health care facilities were inadequate with mental health and care of the elderly only;
- Primary health care workers utilised home visits, scheduling of patient appointments and staff outreach services to improve on quality of their management of primary health services;
- Commitment to primary health care services was significant only with health promotion & education, nutrition education, oral rehydration therapy, immunization services and treatment of common diseases/minor injuries.

## Competing interests

The authors declare no competing interests.
